# Evaluating general practitioners’ focused lung ultrasound competence and findings in patients with suspected community-acquired pneumonia in general practice

**DOI:** 10.1080/02813432.2024.2447083

**Published:** 2024-12-30

**Authors:** Julie Jepsen Strøm, Camilla Aakjær Andersen, Martin Bach Jensen, Janus Laust Thomsen, Christian B. Laursen, Søren Helbo Skaarup, Hans Henrik Lawaetz Schultz, Malene Plejdrup Hansen

**Affiliations:** a Center for General Practice at Aalborg University, Aalborg, Denmark; b Department of Respiratory Medicine, Odense University Hospital, Odense, Denmark; c Odense Respiratory Research Unit (ODIN), Department of Clinical Research, University of Southern Denmark, Odense, Denmark; d Department of Respiratory Medicine and Allergy, Aarhus University Hospital, Aarhus, Denmark; e Department of Cardiology, Section for Lung Transplantation, Rigshospitalet, Copenhagen, Denmark; f Research Unit of General Practice, University of Southern Denmark, Odense, Denmark

**Keywords:** General medicine, ultrasonography, respiratory infections, pneumonia, education

## Abstract

**Objectives:**

To evaluate general practitioners’ (GPs’) ability to perform focused lung ultrasound (FLUS) following a training program and assess FLUS feasibility in general practice. Also, to describe FLUS findings and evaluate GPs’ ability to interpret these in adults with acute lower respiratory tract infection (LRTI) when pneumonia is suspected and assess GPs’ perception of FLUS impact.

**Methods:**

Nine GPs, using point-of-care ultrasound, completed a FLUS training program. Adults (≥ 18 years) with acute cough (< 28 days) and at least one other symptom of acute LRTI, where the GP suspected pneumonia, were subsequently included. All patients received FLUS. The GPs reported FLUS findings, feasibility, and perception of FLUS impact. Recorded FLUS videos from all patients were reviewed by two specialists (Specialist Reference). The specialists assessed FLUS image quality. Agreements between the GPs and the Specialist Reference on FLUS findings were used to evaluate GPs’ ability to interpret FLUS.

**Results:**

Of 91 patients included, FLUS image quality was acceptable or higher in 84 patients (92.4%). FLUS proved feasible with only two scans not completed. The GPs reported FLUS pathological findings in 51.7% of patients in 78% agreement with the Specialist Reference and Cohen’s kappa 0.56. Focal B-lines represented the most frequent pathological findings. The GPs perceived that FLUS impacted change in tentative diagnosis and/or plans for treatment and/or visitation in 29 (32.0%) of patients.

**Conclusion:**

After the training, the GPs performed FLUS well. Interpretation of FLUS pathology presence was of moderate agreement. The GPs perceived that FLUS had impact on patient management.

**Trial registration number:**

ClinicalTrials.gov NCT04711031

## KEY POINTS

- This study found that GPs who were already point-of-care ultrasound users, performed FLUS well following a training program.
- The GPs interpreted presence of pathological findings in about half of patients (51.7%) presenting with symptoms of an acute LRTI, where the GP suspected pneumonia, this in in moderate agreement with FLUS specialists (Cohen’s kappa 0.56).
- The GPs perceived that FLUS impacted change in the tentative diagnosis and/or plans for treatment and/or visitation in 32% of patients.

## Introduction

Acute lower respiratory tract infections (LRTIs) are a significant cause of morbidity and mortality worldwide. Among these infections, bacterial community-acquired pneumonia (CAP) remains a life-threatening condition requiring prompt and accurate diagnosis to guide appropriate treatment and reduce negative outcomes [1]. But differentiating CAP from self-limiting virus infections is often a diagnostic challenge [2,3]. The diagnosis of CAP in general practice relies heavily on the clinical assessment [4], and diagnostic imaging, like chest x-ray, is used only in selected patients [5]. Use of focused lung ultrasound (FLUS) could potentially contribute with real-time imaging as ultrasound is increasingly available in general practice [6–8]. In line with other point-of-care tests, FLUS can provide the general practitioner (GP) with immediate supportive results, making it particularly suitable for use in general practice.

A successful implementation of FLUS in general practice hinges on the adequate training of GPs. Proficiency in FLUS requires not only understanding the sonographic features of pneumonia but also acquiring practical skills in performing and interpreting FLUS. The training needed for GPs to be able to perform and interpret FLUS has not been thoroughly evaluated. So far, only a few studies have described lung ultrasonography training of GPs [9–14], and these have differed much in extent and scope of the training.

Numerous studies have highlighted the high sensitivity and specificity of FLUS for detecting CAP in hospitalised patients, suggesting its potential to improve diagnostic accuracy for LRTIs [15–20]. Small subpleural consolidations (<1-2 cm), multiple and bilateral, with pleural irregularities and bilateral B-lines, typically suggest a viral infection. In contrast, larger single consolidations with air bronchogram, and in some cases pleural effusion, often indicate a bacterial infection. Depending on severity of changes, focal multiple B-lines might be the only observable sign of a bacterial infection, yet they lack specificity [9,21,22]. Regardless, FLUS is unable to identify infections that impact only the deeper regions of the lung and do not extend to the tissue directly under the visceral pleura. Only a few studies have examined the use of FLUS by GPs in general practice for patients suspected of CAP [9,23]. It is crucial to gather more evidence on FLUS in general practice since GPs deal with an unselected patient population and conclude the great majority of contacts without further referral [24]. GPs can be expected to perform FLUS with a lower pre-test probability of pathology and more subtle findings due to the less severe nature of diseases. Consequently, knowledge is needed of what FLUS findings to expect in patients presenting to general practice with symptoms of an acute LRTI when CAP is suspected.

Given that FLUS is an emerging diagnostic tool in general practice, and evidence on its use is sparse [25], little is known about the effect on patient prognosis and potential harms following its use. Hence, long-term follow-up and evaluation of the subsequent patient pathway is also needed.

The objectives of this study was to **1)** evaluate general practitioners’ (GPs’) ability to perform focused lung ultrasound (FLUS) following a tailored training program, **2)** assess FLUS feasibility in general practice and **3)** describe FLUS findings and evaluate GPs’ ability to interpret FLUS in the unselected patient population presenting with symptoms of an acute LRTI in general practice, where the GP suspected community-acquired pneumonia (CAP). Also, we wanted to **4)** assess the GPs’ perception of FLUS impact on management of patients when applying FLUS in this patient population. **5)** We evaluated the patients’ clinical course for 28 days after FLUS was applied through a medical record audit.

## Materials and methods

### Study design

A prospective cohort study with a 28 days’ medical record audit was performed between March 2021 and May 2022. The reporting follows The Strengthening the Reporting of Observational Studies in Epidemiology (STROBE) Statement [26].

### Setting

The study was conducted in nine general practice clinics in Denmark. Almost all inhabitants in Denmark are listed with a GP who act as a gatekeeper for other primary and secondary healthcare providers [27]. At the time of this study 12% of GPs in Denmark were using point-of-care ultrasound (POCUS) [7] but received no reimbursement for related costs (time and equipment).

### Participants

#### Recruitment of GPs and FLUS training program

GPs using POCUS at least once a week in general practice or in out-of-hour services were eligible for participation. No requirements or restrictions were imposed on previous training or use of FLUS. Eligible GPs were recruited through an established GP network at the Center for General Practice at Aalborg University, *via* social media groups for ultrasound in Danish general practice, and through collaboration with the Danish Society for Ultrasound in General Practice (DAUS). Sixteen GPs approached, stating they were willing to participate. As the FLUS training program had economic capacity for only ten participants, a purposely selection of ten GPs was made, to ensure diversity in terms of demographics, POCUS and FLUS experience, organisation of the clinic, and seniority as a GP.

Regardless of previous POCUS and FLUS training and experience, all GPs participated in a FLUS training program prior to enrollment of patients. The content of the training program was in line with the relevant parts of FLUS training courses offered by the Danish and the European Respiratory Societies [28,29]. The purpose of the training program was for the GPs to learn the indications for performing FLUS, to execute the 14-zone FLUS protocol (previously validated in similar clinical contexts in hospital and general practice settings [30–32]) and interpret FLUS findings. The training program consisted of:

1. Theoretical self-studying [33] concluding with a validated theoretical multiple-choice questions (MCQ) test, obtaining an MCQ test score between 0-30. The cut-off for passing was a test score of 20 [34].
2. A one-day hands-on training course completing with a practical assessment of FLUS performance under standardised conditions. The practical assessment was performed with a Lung Ultrasound-Objective Structured Assessment of Ultrasound Skills (LUS-OSAUS) score [35].
3. Between five to ten FLUS scans performed in the GP’s clinic within one month from the hands-on training course. All scans were supervised remotely by specialists in respiratory medicine and FLUS (SHS, HHS, CBL) who all had a FLUS competence level corresponding to EFSUMB level III [36].

#### Recruitment of patients

After completing the training program, GPs were asked to identify eligible patients when they presented to the GP and invite them for participation if they fulfilled the inclusion criteria and none of the exclusion criteria.

##### Inclusion criteria

Adults (≥ 18 years) with acute cough (< 28 days) and at least one other symptom of an acute LRTI (e.g. fever, sputum production, dyspnea, wheezing, discomfort of the chest) where the GP suspected CAP during usual care.

##### Exclusion criteria

- Previous antibiotic prescription for the current episode of acute LRTI.
- Not listed with the GP (no medical record available).
- Uncapable of understanding and signing informed consent.
- Did not wish to participate in the study.

### Study procedure, data collection and outcome measures

#### Patient characteristics

At index consultation, patients initially received usual care as for adults presenting to general practice with symptoms of an acute LRTI. The GPs reported patients’ demographics, comorbidities of relevance, patient-reported symptoms, clinical findings, and results of any point-of-care tests (POCT), e.g. C-reactive protein (CRP), performed as part of usual care.

#### FLUS performance and findings

FLUS was performed as an addition to the GP’s usual care. The GPs used the ultrasonography device already available to them to conduct the 14-zone FLUS protocol [30–32]. The list of ultrasound devices and transducers used is available in supplemental material 1. The GPs used a lung preset if available, otherwise an abdominal preset was used. No ultrasound software was used to detect B-lines or pleural effusion. FLUS pathological findings were predefined (definitions available in supplemental material 2) and dichotomised by the GPs into present or not present for each of the 14 scanning zones.

The GPs made at least one video recording (typically lasting 4-5 sec.) per zone. The 14 recordings constituting one FLUS examination were labelled with scanning zones (Left (L)1-7 and Right (R)1-7). The recordings were exported from the ultrasonography machines in a video format (AVI, WMV or MP4) and collected by the primary investigator (PI) (JJS). After performing FLUS, it was left up to the GPs how to proceed in terms of diagnosis, management and treatment.

#### FLUS assessment

All FLUS video recordings were reviewed independently by two of the FLUS specialists (SHS and HHS). The specialists performed their initial assessment blinded to patient clinical information but were afterwards presented to the clinical information reported by the GP. The specialists were asked if they wanted to change their reporting based on the clinical information and were given the option to do so. Any changes made were tracked.

### FLUS image quality

The FLUS specialists assessed FLUS image quality to assess the GPs’ performance of FLUS under real life conditions. FLUS image quality was rated based on:

- Picture resolution.
- Correct depth and gain.
- Correct placement of the transducer with standard projection (two ribs in a longitudinal axe (bat-sign) and the pleural line horizontal.
- If abdominal organs were present in basal lateral zones.
- If the transducer was kept still.

FLUS image quality was rated on a scale from 1) very low quality and unable to determine potential pathology, to 5) very high quality with images of the same quality as an experienced ultrasound operator would present [37]. In case of disagreement on FLUS image quality between the two specialists, the lowest score was chosen to constitute specialist agreement.

### FLUS findings

The FLUS specialists dichotomised FLUS pathologic findings into present or not present for each scanning zone, in the same manner as the GPs. FLUS findings reported by the two specialists were compared and constituted the ‘Specialist Reference’. A third FLUS specialist (CBL) was consulted in cases of specialist disagreement on findings. The GPs’ findings were compared to the Specialist Reference to calculate the agreement on findings in percentage and unweighted Cohen’s kappa to evaluate the GPs’ ability to interpretate FLUS.

### Outcome measures

#### Objective 1. Evaluation of GPs’ ability to perform FLUS

The ability of GPs to perform FLUS after the training program, was evaluated through the following outcome measures: 1) The practical assessment obtained under standardised conditions during the training program (proportion of maximal possible LUS-OSAUS score), and 2) the proportion of FLUS examinations with acceptable or higher FLUS image quality obtained under real life conditions on included patients.

#### Objective 2. Assessment of FLUS feasibility

Assessment of FLUS feasibility in general practice was based on the following outcome measures, reported by the GPs: 1) number of patients where FLUS was not completed and the reasons for this, 2) time consumption (minutes), and 3) if any unexpected events happened during FLUS. Patient position during FLUS was reported.

#### Objective 3. Description of FLUS findings and evaluation of GPs’ ability to interpret FLUS

FLUS findings were described as 1) Number and proportion of patients with predefined pathological findings present, reported by the GPs and the Specialist Reference. 2) Also, the proportional distribution of pathological findings across the 14 scanning zones was described. The ability of GPs to interpret FLUS after the training program, was evaluated through 3) the agreement on FLUS findings in percentage and unweighted Cohen’s kappa between the GPs and the Specialist Reference.

#### Objective 4. Assessment of GPs’ perception of impact of FLUS

At the end of index consultation, the GPs were asked whether their tentative diagnosis, their confidence in this diagnosis, or their plan for treatment or visitation of the patient changed after FLUS. The GPs also reported if an antibiotic was prescribed. The GPs’ perception of impact of FLUS on management of patients was assessed as the number/proportion of patients where the GP reported change in tentative and/or change in plans for treatment and/or change in plans visitation after FLUS.

#### Objective 5. Evaluation of patients’ clinical course

As a standard care and communication, GPs receive notice of all health-related events, e.g. lab results or discharge notices, and data is automatically uploaded to patients’ electronic medical records.

The PI performed an audit of patients’ medical records up to day 28 after index consultation. Medical records on included patients, including all continuations (all contacts, referrals, discharge letters etc.), were collected on site of the GPs by the PI. The medical records were pseudonymised and transferred to a secure server at Aalborg University. A data collection tool was developed in REDCap and tested before obtaining data on the number/proportion of: 1) patients with any follow-up initiated by the GP, 2) patients referred for secondary ambulatory care, 3) patients with any other imaging performed, and 4) hospitalisations and 5) complications (defined as empyema, lung abscess, pleural effusion, or sepsis).

All data reported by the GPs, were collected in electronic case report forms (e-CRFs) *via* Research Electronic Data Capture© (REDCap©).

### Statistical analysis

Data were analysed using STATA V.17.0 (StataCorp). Categorical variables were summarised using absolute frequencies and proportion and continuous variables using mean and standard deviation (SD) (median and IQR if not normally distributed). Percentage agreement on FLUS findings was calculated as the number of agreements between GPs and the Specialist Reference on pathology present in FLUS for each of the predefined pathological findings, divided by the total number of FLUS. Pathological findings: Any FLUS pathology, ≥ 3 B-lines, Consolidation/Subpleural consolidation, Pleural effusion, Thickened or fragmented pleura. Unweighted Cohen’s kappa was calculated to evaluate the interrater reliability between the GPs and Specialist Reference for the same pathological findings. For interpretation of the kappa values the following criteria were used (< 0.00) poor, (0.00–0.20) slight, (0.21–0.40) fair, (0.41–0.60) moderate, (0.61–0.80) substantial, (>0.80) almost perfect [38].

## Results

### GP characteristics

Nine GPs completed the FLUS training program during January and February 2021, with theoretical MCQ-test scores between 20-26 (median = 25). All GPs obtained remote supervision on FLUS for five to ten patients (median = 7). One GP dropped out between the one-day hands-on training course and remote supervision. This GP did not differ from the remaining GPs regarding MCQ-test or LUS-OSAUS scores. Characteristics of the nine GPs, who completed the training program, are provided in Table 1.

**Table 1.** Characteristics of general practitioners.

	General practitioners (*n* = 9)
Mean age, years (SD)	47.8 (7.0)
Gender^a^	
Male	5 (55.6)
Female	4 (44.4)
Experience as a GP^a^	
< 5 years	4 (44.4)
10-15 years	3 (33.3)
>15 years	2 (22.2)
Experience using POCUS^a^	
< 2 years	3 (33.3)
2-4	3 (33.3)
>4 years	3 (33.3)
Experience using FLUS^a^	
0 years	4 (44.4)
<2 years	2 (22.2)
2-3 years	2 (22.2)
>3 years	1 (11.1)
Location in Denmark^a^	
North Denmark Region	2 (22.2)
Central Denmark Region	3 (33.3)
Southern Denmark Region	2 (22.2)
Region Zealand	1 (11.1)
Capital Region	1 (11.1)
Type of general practice^a^	
Partnership Practice	7 (77.8)
Solo Practice	2 (22.2)

^a^Values presented as n (%).

SD: Standard deviation; GP: General Practitioner; POCUS: Point of care ultrasound; FLUS: Focused lung ultrasound.

### Patient characteristics

**Figure 1.**
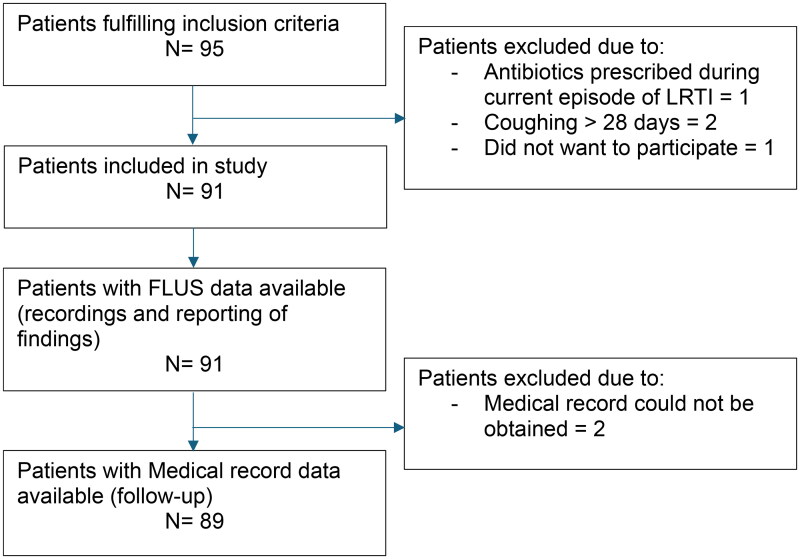
Diagram showing flow of patients in the study. LRTI: Lower respiratory tract infection; FLUS: Focused lung ultrasound.

Between March 2021 and May 2022, the GPs included a median of 9 patients each (min. 6, max. 25). FLUS recordings and data from index consultation were available for analysis for all 91 patients included. Data from medical records were available for 89 patients. Figure 1 illustrates patient flow. Characteristics of patients included are provided in Table 2.

**Table 2.** Characteristics of patients.

	Patients (*n* = 91)
Gender^a^	
Male	39 (42.9)
Female	52 (57.1)
Age (years)^b^	56.3 (15.9)
Comorbidities of relevance^a,d^	
None	49 (53.9)
Lung disease	29 (31.9)
Heart disease	8 (8.8)
Other	14 (15.4)
Patient-reported symptoms^a^	
Sputum production	65 (71.4)
Dyspnoea	47 (51.7)
Chest discomfort	46 (50.6)
Fever (tp. ≥ 38.0 °C)	40 (44.0)
Poor general well-being	29 (31.9)
Other	15 (16.5)
Clinical findings^a^	
Abnormal lung auscultation	32 (35.2)
Poor general appearance	12 (13.2)
Sputum production	9 (9.9)
Other	11 (12.1)
None	8 (8.8)
Vital signs	
Increased respiratory rate^a,e^	7 (7.7)
Respiratory rate (breaths/min.)^c^	21.5 (20-24)
O_2_ saturation performed^a^	47 (51.7)
O_2_ saturation (%)^c^	97 (95-98)
Heart rate measurement performed^a^	20 (21.9)
Heart rate (beats/min.)^c^	83.5 (72.5-94.5)
Point of care tests (poct)	
CRP poct performed^a^	79 (86.8)
CRP level (mg/L)^c^	12 (5-42)
Leucocytes poct performed^a^	13 (14.3)
Leucocytes (10^9^/L)^c^	7.5 (7-8.2)
Other^a^	5 (5.5)
None^a^	9 (9.9)
Antibiotics prescribed^a^	34 (37.4)

^a^Values presented as n (%).

^b^Values presented as mean (SD).

^c^Values presented as median (IQR). Values based on the number of patients with the poct performed.

^d^Comorbidities perceived of possible relevance to LRTI severity or FLUS findings by the GP.

^e^Increased respiratory rate ≥ 20 breaths/min.

SD: Standard deviation; LRTI: Lower respiratory tract infection; FLUS: Focused lung ultrasound; IQR: Interquartile range; CRP: C-reactive protein.

### Evaluation of GPs’ ability to perform FLUS (objective 1)

**Figure 2.**
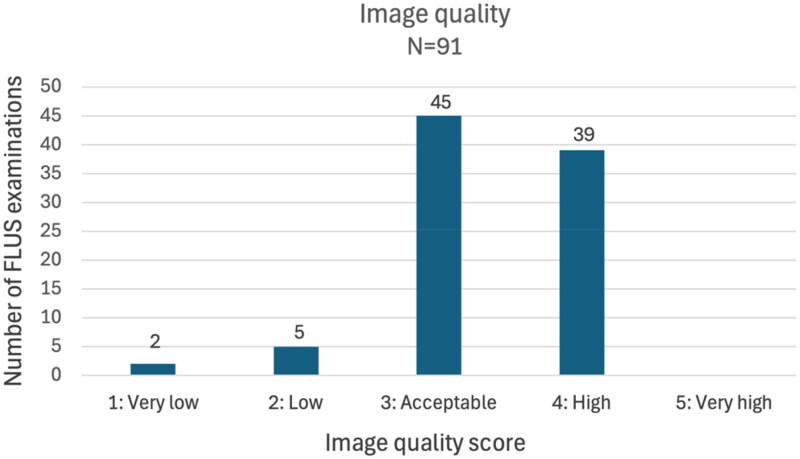
FLUS image quality. FLUS: Focused lung ultrasound.

The GPs obtained LUS-OSAUS scores between 96% to 100% of max. score (median = 99%). FLUS image quality was rated acceptable or higher in 84 (92.4%) of patients (Figure 2).

### Assessment of FLUS feasibility (objective 2)

FLUS was completed in all patients, except two, where FLUS was only partly performed due to logistical reasons. Time consumption for performing FLUS (including labelling and recording of videos) was a median of 15 min (IQR 10-15) but varied from 3 to 30 min. No unexpected events during FLUS were reported. About half of patients (54%) were in a supine position for zones 1-4 and in a sitting position for zones 5-7. The remaining patients were sitting upright during the full FLUS examination.

### Description of FLUS findings and evaluation of GPs’ ability to interpret FLUS (objective 3)

**Figure 3.**
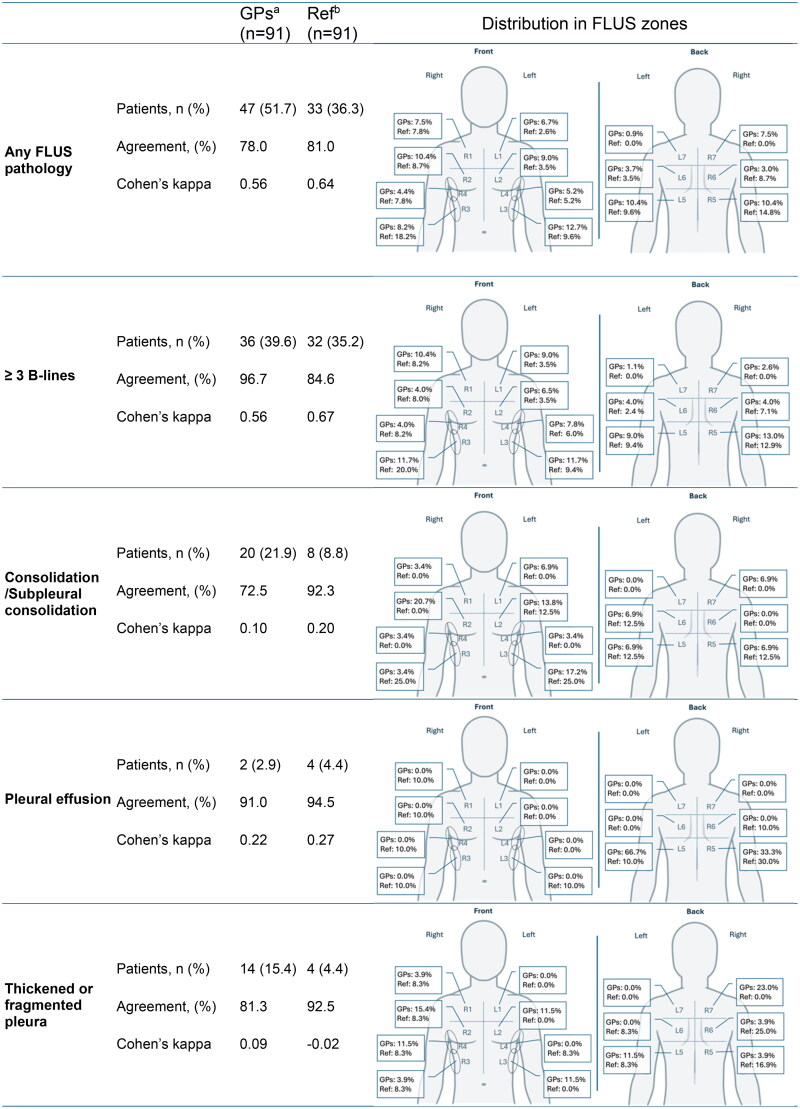
FLUS findings in patients (*n* = 91) and agreement by the GPs and Specialist Reference (Ref). ^a^Agreement and Cohen’s kappa: Agreement between the GPs and the Specialist Reference (Ref) ^b^Agreement and Cohen’s kappa: Agreement between the FLUS Specialists (SHS and HHS) FLUS: Focused lung ultrasound; GPs: General Practitioners; Ref: The specialist reference.

FLUS findings, agreements on findings between GPs and Specialist Reference and distribution of findings across the 14 FLUS zones are illustrated in Figure 3.

GPs interpreted presence of pathological findings in about half of patients (51.7%) in moderate agreement with the Specialist Reference (Cohen’s kappa *k* = 0.56). The most common findings were focal pathologic B-lines (≥ 3 B-lines in one zone) also detected in moderate agreement with the Specialist Reference followed by consolidation/subpleural consolidation. The GPs and Specialist Reference agreed on two consolidations being with the sonographic characteristics of a pneumonic consolidation (see supplemental material 2) and the rest of consolidations/subpleural consolidations were unspecific. As frequency of findings declined, so did in general the agreement between GPs and the Specialist Reference, but also the agreement between the two FLUS specialists (Figure 3). Overall, the GPs reported more pathology to be present than the Specialist Reference, apart from pleural effusion and interstitial syndrome. Interstitial syndrome (≥ 3 B-lines in ≥ 2 zones in each hemithorax) (not shown in Figure 3) was reported in two patients (2.2%) by the GPs and in five patients (5.5%) by the Specialist Reference. The agreement between GPs and the Specialist Reference regarding interstitial syndrome was 92.3% and *k* = 0.27. The agreement between the two FLUS specialists regarding interstitial syndrome was better with an agreement of 96.7% and *k* = 0.56. Pneumothorax was not detected in any patients. One GP accounted for over a quarter of the examinations (25 patients); however, excluding this GP did not significantly alter agreement on any FLUS pathology and focal B-line pattern (≥3 B-lines) (see supplemental material 3 for sensitivity analysis). Agreement on consolidation and pleural effusion, did however decline and agreement on pleural thickening/fragmentation increased when excluding this GP from analysis.

The specialists did not in any case change their assessment and reporting of FLUS findings after clinical information was revealed.

### GPs’ perception of impact of FLUS on patient management (objective 4)

The GPs’ perception of FLUS’ impact on their tentative diagnosis, confidence in the tentative diagnosis, and influence on plans for patient treatment or visitation are listed in Table 3. The GPs perceived that FLUS changed the tentative diagnosis and/or plans for treatment and/or plans for visitation in 32% of patients.

**Table 3.** GPs’ Perception of FLUS impact.

	Patients^a^ (*n* = 91)
Tentative diagnosis for this patient changed after FLUS	19 (20.9)
Plans for treatment of this patient changed after FLUS	23 (25.3)
Plans for visitation of this patient changed after FLUS	10 (11.0)
Overall perception of change^b^	29 (32.0)
More certain of my diagnosis for this patient after FLUS	69 (75.8)

^a^Values presented as n (%).

^b^Overall perception of change include change in tentative diagnosis and/or plans for treatment and/or plans for visitation.

### Evaluation of patients’ clinical course (objective 5)

The GPs initiated follow-up, such as for example re-consultation, phone call, repeated FLUS or blood tests, in 36 (40.4%) of the 89 patients with 28-day follow-up data available. Twenty patients (22.5%) had other imaging than FLUS performed during the follow-up period and four patients (4.5%) were referred for secondary sector ambulatory care. The seven patients (7.9%) who were hospitalised during follow-up and the one patient (1.1%) developing a complication, all had antibiotics prescribed at index consultation.

## Discussion

### Main findings

The GPs had a high ability to perform FLUS following the tailored training program. FLUS proved feasible, with only two scans not fully completed due to logistical reasons and no unexpected events happened. Time spent on performing FLUS was however extensive (median 15 min.), but also included labelling and recording of videos. Pathological findings were found in about half of patients, with a focal B-lines pattern being the most common pathology reported. Pathological findings were distributed in all 14 FLUS zones but seldom present in zones 7 (L7 and R7). The GPs’ ability to make the overall interpretation if any FLUS pathology was present, was of moderate agreement with the Specialist Reference (*k* = 0.56).

Importantly, the GPs perceived that FLUS changed either their tentative diagnosis and/or plans for treatment and/or visitation in 29 (32.0%) of patients.

### Strengths and limitations

One strength of this study is the alignment of the provided FLUS training program with courses offered by the Danish and European Respiratory Societies. Additionally, the program was tailored to the general practice setting in collaboration with leading respiratory medicine FLUS specialists. The program included both validated theoretical and practical assessments, with practical skills evaluated under both standardised and real-life conditions.

Another notable strength is the independent blinded review of all FLUS examinations by two specialists, providing important interrater agreement data for evaluating GP-Specialist agreement.

However, when interpreting the results, the variability in the number of patients included by each GP (range: 6-25) must be considered. One GP accounted for over a quarter of the examinations (25 patients) This variation influences the consistency in abilities to interpretate FLUS among the GPs. Excluding the highest recruiting GP from analysis (sensitivity analysis) did affect the agreement with the Specialist Reference on the least frequent pathologic findings, but in both directions (consolidations, pleural effusion and pleural thickening/fragmentation) (supplemental material 3). Only nine GPs participated in this study and given their use of POCUS, they most likely constitute a selected group of GPs, which might influence the generalizability of the study to a broader population of GPs.

Although image quality was deemed acceptable in 92.4% of examinations, differences in image resolution between the ultrasound device screens from which the GPs reported their findings and exported video formats could impact agreement. This could be of particular importance in cases where findings were delicate and subtle. GPs may also have detected and reported FLUS pathology that they mistakenly did not capture during their 4-5 s of recording. This could again explain the GP’s higher pathology reporting compared to the specialists.

Data on the exact duration of patients’ symptoms prior to the consultation was not collected. The duration of symptoms could potentially affect FLUS findings and/or POCT values if the duration of symptoms was very short (< 24 h) or extensive (close to 28 days).

### Comparison to existing literature

The overall proportion of patients with any FLUS pathology and the proportion with focal B-lines, are in line with the findings in the only previous study, to our knowledge, describing FLUS findings in a comparable population and setting [9]. We found, however, consolidations/subpleural consolidations in a smaller proportion of patients than in the study by Rodríguez-Contreras et al. who included both adults and children suspected of having CAP. More patients in that study presented with a fever and abnormal lung auscultation compared to ours, suggesting more patients in our study having a viral infection or being less severely ill. This assumption is also supported by the median CRP value in the present study being only 12 mg/L (IQR 5-42) in the patients who had a CRP poct performed. A difference in the etiology of infection, extent of illness, or severity could explain the smaller proportion of consolidations found in our study.

The kappa coefficient of overall agreement between GPs and the Specialist Reference on the presence of any FLUS pathology found (*k* = 0.56) is not quite as high as for the overall interrater-agreement on FLUS findings by GPs in children suspected of pneumonia in a low-resource setting found in a Peruvian study (*k* = 0.65) [13]. Ellington et al. however, looked at an interrater agreement between three GPs performing and interpreting FLUS and did not compare to a specialist reference. Moreover, the GPs had received a seven days’ FLUS training program. A previous study by Pietersen et al. did compare FLUS agreement performed by frontline primary care personnel with a specialist reference, but with FLUS being performed by emergency medicine services personnel. The agreement on any pathology present in that study was 87.7% and *k* = 0.44 [37].

The FLUS training program used in the present study is highly comparable to a training program evaluated for primary care physicians engaged in COVID-19 outpatients’ evaluation, also comprising theoretical self-paced learning, practical hands-on training, and supervision on real-life FLUS [12]. The same accounts for a Danish study training primary care acute nurses for in-home FLUS assessments of geriatric patients [39]. More comprehensive FLUS training, limited to the diagnosis of pediatric pneumonia, has been described for GPs with a 7 days’ didactic and practical training by one group of expert trainers and followed by direct supervision by local experts on 25 scans [11]. That training program demanded much higher resources but also resulted in high accuracy and higher agreements (*k* = 0.8-0.9) compared to ours. The high level of accomplishment was also maintained over time in the study by Pervaiz et al. which is an aspect that remains unstudied in ours.

Our results indicate that the time needed for performing FLUS (even when time spent was including labelling and recording) is what could question the feasibility of FLUS in general practice. This indication is supported by a study exploring the acceptability of lung ultrasound in GPs [40]. FLUS duration proved less of an issue in a feasibility study in general practice with a median FLUS duration of four minutes, but this being with only an 8-zone protocol [14]. Another barrier previously highlighted, is GPs not trusting their own interpretation of ultrasonography images [40]. This barrier was found to be less pronounced for GPs using POCUS also for other purposes than FLUS. The effect of also using POCUS for other purposes is perhaps what is being reflected in our study, where the GPs (already POCUS users) perceived a high impact of FLUS on their management of patients, indicating a certain amount of confidence in their own findings and interpretation.

### Implications for research and practice

The study shows that FLUS in patients suspected of CAP in Danish general practice was perceived by the GPs to impact the handling of a significant proportion of patients. Implementing FLUS in general practice as part of usual care for patients suspected of CAP may reduce the diagnostic uncertainty and possibly reduce unnecessary antibiotic prescribing. A randomised controlled trial has been initiated determining whether adults who present with symptoms of an acute LRTI in general practice - and who have FLUS performed in addition to usual care - are treated less frequently with antibiotics than those who receive only usual care [41].

## Supplementary Material

Supplemental material 3.docx

Supplemental material 2.docx

Supplemental material 1.docx
