# Supplementary material for: Evaluating general practitioners’ focused lung ultrasound competence and findings in patients with suspected community-acquired pneumonia in general practice
Source: Scand J Prim Health Care. 2024 Dec 30;43(2):359–69. doi: 10.1080/02813432.2024.2447083 (PMC12090303; doi:10.1080/02813432.2024.2447083)
Supplement: Supplemental material 3.docx [file IPRI_A_2447083_SM8182.docx]

Supplemental material 3:

a)

Percentage agreement and unweighted Cohen’s kappa between GPs and the Specialist Reference when the GP including most patients (25 patients) excluded (outlier GP).

| Outlier GP excluded (N=66) | % Agreement | Cohen’s kappa |
| --- | --- | --- |
| Any FLUS pathology | 77.3 | 0.56 |
| ≥ 3 B-lines | 86.4 | 0.67 |
| Interstitial syndrome | 93.9 | 0.19 |
| Consolidation/Subpleural consolidation | 77.3 | -0.04 |
| Pleural effusion | 92.9 | -0.02 |
| Thickened or fragmented pleura | 81.8 | 0.16 |

b)

Percentage agreement and unweighted Cohen’s kappa between GPs and the Specialist Reference when FLUS with lower than acceptable image quality (n=7) excluded.

| FLUS with lower than acceptable image quality excluded (N=84) | % Agreement | Cohen’s kappa |
| --- | --- | --- |
| Any FLUS pathology | 77.4 | 0.51 |
| ≥ 3 B-lines | 86.9 | 0.72 |
| Interstitial syndrome | 90.5 | 0.08 |
| Consolidation/Subpleural consolidation | 71.4 | 0.11 |
| Pleural effusion | 92.2 | 0.26 |
| Thickened or fragmented pleura | 81.0 | 0.09 |
