# Supplementary material for: Evaluating general practitioners’ focused lung ultrasound competence and findings in patients with suspected community-acquired pneumonia in general practice
Source: Scand J Prim Health Care. 2024 Dec 30;43(2):359–69. doi: 10.1080/02813432.2024.2447083 (PMC12090303; doi:10.1080/02813432.2024.2447083)
Supplement: Supplemental material 2.docx [file IPRI_A_2447083_SM8181.docx]

Supplemental material 2: Definitions of FLUS pathologic findings.

*FLUS pathologic findings*

FLUS pathologic findings were predefined, and the GPs were trained in recognizing and defining possible pathologic findings during the FLUS training program. The definitions of FLUS findings were based on the European Federation of Societies for Ultrasound in Medicine and Biology (EFSUMB) coursebook^1^ and International evidence-based recommendations for point-of-care lung ultrasound^2^.

1. Any FLUS pathology: Presence of any of the below mentioned (b-i)
2. ≥ 3 B-lines: Multiple (≥3) B-lines in at least one zone.

B-lines: Laser-like vertical echogenic artefacts arising from the pleural line, spreading without fading to the edge of the screen and moving synchronously with lung sliding.

1. Interstitial syndrome: Multiple (≥3) B-lines in at least 2 zones on each hemithorax present.
2. Consolidation: Loss of aeration, which allows visualization of the lung parenchyma with sonomorphologic characteristics that resemble a solid organ or tissue. Pathognomonic for a pneumonic consolidation is the presence of air-bronchograms and serrated or blurred margins.
3. Subpleural consolidation: Small subpleural consolidation between 2 and 20 mm in size that moves together with lung sliding.
4. Pleural effusion: Anechoic or hypoechoic space between the visceral and parietal pleura.
5. Pleural thickening or fragmentation: Hypoechogenic thickening of the pleura with a rough appearance and interruption of the normally smooth pleura.
6. Pneumothorax: Area without lung sliding, lung pulse or B-lines, with the presence of a lung point in an adjacent area.
7. Other FLUS pathology: Other incidental findings by FLUS were described according to the ability of the GPs.

^1^ Mathis G et al. EFSUMB Course Book, 2nd Edition. Ultrasound of the Chest.

<https://efsumb.org/wp-content/uploads/2023/07/ECB2nd_-chest_FULL.pdf>

Accessed august 15th 2024.

^2^ Volpicelli G et al.; International Liaison Committee on Lung Ultrasound (ILC-LUS) for International Consensus Conference on Lung Ultrasound (ICC-LUS). International evidence-based recommendations for point-of-care lung ultrasound. Intensive Care Med. 2012 Apr;38(4):577-91.
