# Supplementary material for: Evaluating general practitioners’ focused lung ultrasound competence and findings in patients with suspected community-acquired pneumonia in general practice
Source: Scand J Prim Health Care. 2024 Dec 30;43(2):359–69. doi: 10.1080/02813432.2024.2447083 (PMC12090303; doi:10.1080/02813432.2024.2447083)
Supplement: Supplemental material 1.docx [file IPRI_A_2447083_SM8180.docx]

Supplemental material 1:

Ultrasound machine systems and transducers used for FLUS by the GPs.

| **Ultrasound machine system** | **Transducer used** |
| --- | --- |
| Butterfly iQ | Butterfly iQ |
| GE Logic V2 | Convex (abdominal) |
| GE Logiq V2 | Convex (abdominal) |
| GE Logiq P9 | Convex (abdominal) |
| Siemens P500 FROSK | Convex (abdominal) |
| Sonoscape X3 | Convex (abdominal) |
| Sonoscanner Orcheo Lite | Convex (abdominal) |
| My Lab gamma | Convex (abdominal) |
| Vinno E10 | Convex (abdominal) |
